# Addressing Disparities in Diabetes Management Through Novel Approaches to Encourage Technology Adoption and Use

**DOI:** 10.2196/diabetes.6751

**Published:** 2017-07-13

**Authors:** Amy R Sheon, Shari D Bolen, Bill Callahan, Sarah Shick, Adam T Perzynski

**Affiliations:** 1 Urban Health Initiative School of Medicine Case Western Reserve University Cleveland, OH United States; 2 Center for Health Care Research and Policy Department of Medicine MetroHealth/Case Western Reserve University Cleveland, OH United States; 3 Connect Your Community Cleveland, OH United States

**Keywords:** diabetes, chronic illness, vulnerable populations, digital divide, community health workers, healthcare disparities, patient portals, patient engagement, meaningful use, health literacy

## Abstract

Type 2 diabetes (T2D) is one of the nation’s leading drivers of disability and health care utilization, with elevated prevalence among individuals with lower education, income, and racial/ethnic minorities. Health information technology (HIT) holds vast potential for helping patients, providers, and payers to address T2D and the skyrocketing rates of chronic illness and associated health care costs. Patient portals to electronic health records (EHRs) serve as a gateway to consumer use of HIT. We found that disparities in portal use portend growing T2D disparities. Little progress has been made in addressing identified barriers to technology adoption, especially among populations with elevated risk of T2D. Patients often lack digital literacy skills and continuous connectivity and fear loss of the relationship with providers. Providers may experience structural disincentives to promoting patient use of HIT and apply hidden biases that inhibit portal use. Health care systems often provide inadequate training to patients and providers in use of HIT, and lack resources devoted to obtaining and optimizing use of data generated by HIT. Lastly, technology-related barriers include inadequate consideration of user perspectives, lack of evidence for patient-focused apps, and lack of features to enable providers and health care systems to readily obtain aggregate data to improve care and facilitate research. After discussing these barriers in detail, we propose possible solutions and areas where further research is needed to ensure that individuals and health care systems obtain the full benefit of the nation’s planned $38 billion HIT investment. A digital inclusion framework sheds new light on barriers posed for patients with social health inequalities. We have determined that partnerships with community organizations focused on digital inclusion could help health systems explore and study new approaches, such as universal screening and referral of patients for digital skills, health literacy, and Internet connectivity.

## Introduction

### The Promise of Health Information Technology for Type 2 Diabetes Management

More than 29 million adults have type 2 diabetes (T2D), with prevalence elevated among Hispanics (12.8%), blacks (13.2%), and individuals with less than a high school education (13.6%) versus some college (7.8%) and whites (7.6%) [1]. Medication, diet, and physical activity can limit the health consequences of T2D [2,3], but recommended targets for blood sugar, lipids, and blood pressure control are met by only about one-half of those affected [4].

Responding to federal financial incentives [5,6], physicians rapidly adopted electronic health records (EHRs) between 2009 and 2015, making portals available for 65% of patients to view their records [7]. Serving as persuasive technology [8], portals offer significant potential for improving medical management of chronic diseases. Portals also open the door to telehealth and to remote monitoring of data from connected devices such as glucose monitors [9]. Remote glucose monitoring saves California Medicaid $939 yearly per diabetic patient. Nearly every state Medicaid program now covers some telehealth and about one-half reimburse for remote monitoring [10]. Barriers to technology adoption among populations facing social health inequalities (SHIs) [11], however, are not being addressed and research is not keeping pace.

### Digital Inclusion Perspective on Health Information Technology

MetroHealth System in Cleveland, Ohio, was the nation’s first public hospital to adopt EHRs (1999-2002) [12]. In 2015, MetroHealth received a national award for improving care while returning nearly $8 million on its health information technology (HIT) investment [13]. Nevertheless, similar to adoption rates reported elsewhere [14-16], only 29.1% (70,835/243,248) of MetroHealth adult patients have logged in to their portal accounts. Use lags for blacks (23.4%) and Hispanics (23.8%) and for those without commercial insurance (39.3%) [17]. Many patient barriers to portal adoption [18] are also associated with the digital divide.

Based on engagement with the digital inclusion movement, we offer this Viewpoint to illuminate HIT adoption barriers faced by those with reduced digital skills and health literacy and those who lack always-on smartphones and ample data plans. Digital inclusion refers to “the activities necessary to ensure that all individuals and communities, including the most disadvantaged, have access to and use of information and communication technologies” [19]. We suggest new approaches to addressing HIT-related disparities in hopes of defying the inverse care law [20].

## Findings

### Patient-Related Barriers to Health Information Technology Adoption

Some leading barriers to adoption among patients include inadequate Internet access, digital skills, and eHealth literacy and concerns about diminution of the relationship with the provider. General technology adoption among SHI populations is a prerequisite for HIT adoption. Differences in residential access to broadband explained 68% of portal use variation among MetroHealth patients [17]. Lack of computer skills and Internet access have been identified by others as barriers to portal use [21-24].

Nationally, census data show that home broadband Internet subscriptions are considerably less prevalent among seniors (67.0%), blacks (69.7%), Hispanics (74.5%), and those with annual income less than $20,000 (48.8%) compared with all households (80.8%) [25]. Smartphone adoption is growing rapidly but gaps remain for those over 65 years (47.1%), with less than high school (50.9%) and high school (62.8%) education, with a disability (51.4%), and living in poverty (60.2%) compared with 74.8% ownership for all individuals age 15 years and older. Ownership among Hispanics (75.8%) slightly exceeds that for whites (74.6%); blacks lag only slightly (70.3%) [26].

Smartphone access to portals is new and not widespread [17,27], but apps are sometimes easier to use than Web-based services. However, mobile Internet is generally slower and more expensive than fixed broadband. Dependency on smartphones for Internet is now seen among 13.9% to 18.5% of SHI groups versus 7.9% of all adults [26]. About half of smartphone-dependent individuals report needing to disconnect service at times [28]. Among low-income mobile broadband subscribers, 30% exceed their data caps every month, resulting in service interruption (21%), slowed speeds (24%), or costly fees (27%) [29]. Data disruptions are especially problematic for enabling patients to address underlying social determinants of health in light of Internet-based job applications, government benefits management, coursework requirements, and the like. Computers offer large screens and keyboards that are easier for typing and searching. Fixed broadband connections are typically fast, secure, and include ample data. However, 59% of nonusers cite cost as the leading barrier to adoption [30]. Having both mobile and fixed connectivity optimizes convenience and productivity but is an unaffordable luxury to many.

Cellular phones (smart or not) are replacing landlines, especially among adults living in poverty, those younger than 44 years, and Hispanics. Cost-related cellular service interruptions are particularly disruptive to mobile-only households. Portals provide a vital connection with health care providers for those in a state of “dependable instability” of mobile communications [31].

Some evidence shows benefits from home monitoring of glucose, blood pressure, and weight [32]. However, only 20% of invited participants agreed to join a study in England of telehealth with regular transmission of physiologic information; concerns about operating the technology were an important barrier [33].

A systematic review of adherence-focused mobile apps found improvements in diabetes-specific clinical outcomes in 11 of 26 randomized trials [34]. Use of health-related smartphone apps is prevalent although user characteristics are inconsistent across surveys [35,36]. Portal adoption is strongly predicted by eHealth literacy [16,37,38], the ability to obtain and use health information from digital devices [39,40]. More common among those age 65 years and older, blacks and Hispanics, and individuals with low income and education [41], lower health literacy is associated with requiring more assistance and time to perform standard portal tasks [42].

MetroHealth patients had small differences by race or type of insurance in viewing lab results but larger differences using functions that required composition such as requesting advice and responding to messages. Patients were less likely to send messages if black, Hispanic, not commercially insured, less educated, or with less access to broadband [17]. SHI disparities in messaging frequency have also been reported [15,16].

Diabetic patients [43] and black and Latino portal nonusers [44] revealed in surveys and focus groups fears that portals would undermine relationships with providers or reduce valued human contact. Others reported fears about portal use invoking government surveillance and deportation [45].

### Provider- and Health Care System–Related Barriers to Health Information Technology Adoption

Providers may encounter structural disincentives or act on biases that inhibit patient use of HIT. Provider endorsement is important for portal adoption among diabetic [21] and other patients [16]. However, providers may withhold portal promotion fearing income loss from reduced clinical visits [46,47] or lacking time [34] or compensation [45] for responding to messages.

About 40% of adult MetroHealth patients had established portal accounts by 2015 [13], but only 29.1% (70,835/243,248) had ever logged in [17]. This gap has been reported elsewhere [14,48,49], perhaps reflecting an observation from a safety net facility study: “The assumption built into the [Meaningful Use] metric is that providing patients with instructions is sufficient to convert them into active portal users. Therefore, outreach strategies often emphasized enrolling as many patients as possible” [45].

Provider bias in recommending portals has received little attention. According to results of a national survey, the odds of a provider offering portal access were only 0.59 for blacks (CI 0.42-0.84) versus whites and 0.47 for Hispanics (CI 0.32-0.68) versus non-Hispanics [50]. Similar findings were reported among federally qualified health center patients [22].

Portal recommendations may reflect provider assumptions about whether patients have digital skills or value connectivity. Reminiscent of historic discriminatory lending practices [51], Callahan and the National Digital Inclusion Alliance recently documented “digital redlining” by the predominant Internet Service Provider in Cleveland, who “withheld fiber-enhanced broadband improvements” from areas with high concentrations of black and low-income residents [52]. This pattern was also reported for the entire state of California [53]. Thus, patients facing SHIs may lack the option of home broadband at any price.

Among nearly 50,000 patients with T2D seen at health systems participating in the Better Health Partnership regional health quality improvement collaborative (for which Bolen serves as the Director of Cardiovascular Disease Programs), careful analysis of aggregate EHR data led to improvements and reduced disparities in care and outcomes [54]. Yet health care systems may lack resources that enable optimal use of the EHR for such purposes or for direct patient engagement. Expanded portal use could generate data that would add value to EHR data, especially when typologies of portal usage patterns are applied [55].

### Technology Barriers

HIT developers have been long criticized for lack of user-friendly design [56,57]. Usability issues were the main reason for nonuse of HIT among low-income racial and ethnic minorities [58]. Little has been written about how health systems support patient use of portals, suggesting that little help is being offered. Portal research study publications usually note that instruction was provided by clinicians or research assistants; such individuals are unlikely to have expertise in digital skills training. Password management and recovery is challenging for those with lower digital literacy [43] and “even for extremely experienced users with a high degree of savvy regarding new technologies” [58]. Safety net patients who were regular Internet users had difficulty registering for the Diabetes Prevention Program mobile app because they “rarely checked email and some participants did not have email accounts, requiring help to set up new ones.” Patients with fewer computer skills had fundamental difficulties navigating an online form, such as knowing how to enter a Web address or skip a question [59]. Patients reliant on public Wi-Fi or shared phones are especially concerned about account security [43,60].

Much consumer-focused HIT, including patient portals, has been introduced with limited evidence of effectiveness. Regarding diabetes-specific outcomes, a 2014 review found consistent improvements in hemoglobin A_1c_ levels but not in other biomarkers [61]. In another study, individuals randomized to use wearable activity trackers and a Web interface to monitor diet lost *less* weight than those in the control arm [62]. Nonetheless, some evidence shows that with heavy utilization portals can have an impact on SHI populations [63,64].

## Solutions

Low-cost smartphones, Internet access programs, and free digital skills training are now widely available [65], offering an unprecedented opportunity to address key portal use barriers. Four actions to reduce SHIs through eHealth have been suggested: promote universal access to eHealth technology, consider patient literacy level, consider cultural factors, and engage populations at risk of SHIs with eHealth design [11]. We offer additional suggestions for expanding HIT adoption.

The MetroHealth Patient Centered Media Lab team is testing having physicians issue prescriptions for portal adoption and offering training for the portal smartphone app. Elsewhere, a portal opt-out approach enabled community health centers to reduce racial disparities in repeat portal use [66]. To go even further, we suggest screening all patients for digital skills (with a checklist, hands-on demo, or free online tools) [67,68]; health literacy (with a single question) [69,70]; and connectivity (using standard survey questions) [30,71,72]. Or patients could simply be asked about their interest in low-cost desktop, tablet, and mobile equipment and broadband or mobile data and referred to local partners for assistance. The Centers for Medicare and Medicaid Services funded the United Way of Greater Cleveland and several other communities to screen patients for certain barriers to health such as lack of funds for transportation and utilities and refer them to community organizations for support [73,74]. Screening for Internet access and then referring clients to community partners for skills training and connectivity would be a valuable augmentation to this new initiative.

As part of a national program [75,76], Callahan engaged community organizations in several cities (from 2010 to 2012) to equip, train, and connect 21,000 low-income residents who lacked computers or home Internet. Training and connectivity support were transformative for many [77,78], and we are now referring patients to that initiative’s Cleveland-area partners for skills training and connectivity support. Patients may be eligible for free or reduced-cost equipment and service through federal, state (including Medicaid), and commercial programs [65,79,80]. (Plans with unlimited data are especially valuable versus ones with low data caps [29]). Similar partnerships are underway in just a few other cities [81-85]. However, integration of clinical and community systems is now seen as essential for treating obesity and related chronic diseases [86]. Community Reinvestment Act funds invested by local banks in communities to redress the legacy of discriminatory lending from the 1930s to the 1970s could be leveraged to expand broadband access and skills training [87].

Consumer-focused software should not require instruction. However, between digital skill and health literacy gaps and technology shortcomings, portal training is essential for increased adoption, reduced disparities, and increased impact on health. Instruction time will vary from 15 minutes among eHealth literate individuals [83,88,89] to many hours for patients with such conditions as serious mental illness [90]. Portal training could be tailored based on digital skill and health literacy assessments; options include on-line, tutor-facilitated, individual, and group classes [82]. Portal training should use evidence- or theory-based techniques geared to the pivotal moments in the learning progression, from fear to mastery [91].

Tieu [42] identifies 5 key portal functions on which patients should be trained: logging in, viewing visit summaries, viewing prescribed health education information, viewing test results, and looking up information in a connected online library. Those with low numeracy may need assistance interpreting laboratory results [92]. Dictation and autocomplete [60] plus template messages could help with tasks requiring composition. Portal training should address concerns around authentication and password management, data security, loss of the personal relationship with the provider, and fear of deportation [45] as expressed by persons with SHIs.

In preparation for expanding referrals of patients to the portal, we are training community health workers to perform digital skills assessments and some portal training; others are using technology navigators for similar purposes [48]. Clinical and digital skills training content should be provided by individuals with the relevant expertise. Family caregivers represent a largely untapped resource to help patients bridge digital skill and connectivity gaps, albeit raising privacy concerns [93].

## Discussion

### Further Research and Development

To accelerate health improvement and reduced disparities through HIT, better technology and intervention studies are needed. Extracting the full value from rich EHR and portal data requires dedicated, trained staff [94]. Their jobs could be made much easier if software makers included tools for such purposes.

Mobile access could increase portal use, but there has been little uptake at our own or other institutions [17,27]. Portal instructions and health information should be presented at patients’ reading level, with ready access to more detailed or simplified information. Movies, illustrations, and graphs are especially useful for those with lower literacy or language barriers [18]. Portal adoption interventions must be developed and tested. The Network of Digital Evidence in Health (NODE Health) is applying evidence-based medicine rigor to address the current void in much digital health technology [95]. As a NODE Health consortium member, Sheon seeks to ensure [96] that overlooked SHI perspectives [97] are considered in assessment of digital health efficacy [98].

Patient input to technology development benefits both underserved and advantaged patients [58]. Inclusion of technology novices is especially important for usability testing [57]. One project paid “citizen scientists” for helping to create a diabetes mobile app [99]; SHI patients may need such funds to participate. For patient convenience, treatment recommendations that are shared among comorbidities, such as physical activity for T2D and depression, could be addressed in a single app or portal feature [57]. In the Patient Centered Media Lab, Perzynski [100] engages patients in designing and deploying HIT apps such as an augmented reality exercise game to prevent T2D. Perzynski and Shick [101] have created a single-click app that displays social and environmental determinants of health specific to a patient’s residential address plus links to community support to address these issues.

Finally, mobile phone ownership is almost universal in the United States with disparities nearly closed [102]. Short message service (text) messaging improves insulin titration [103] and medication adherence for chronic diseases [104]. Text messages require only a cellular phone and do not require a data plan and should thus be considered for interventions.

### Conclusion

A digital inclusion lens reveals digital skill and connectivity barriers that must be addressed to avoid widening T2D disparities. Health care systems should partner with local digital inclusion advocates to screen and help patients obtain low-cost Internet service, equipment, and basic digital skills training. These are essential for portal training to be efficient and effective. Portal training should be informed by those with expertise in digital skills and health literacy acquisition. Paraprofessionals such as community health workers could be trained accordingly to assume some of these responsibilities at a relatively low cost. Research on the cost effectiveness and impact of these novel approaches should lead to support for broad dissemination, if not insurance reimbursement.

## Abbreviations

eHealth: electronic health
EHR: electronic health record
HIT: health information technology
NODE Health: Network for Digital Evidence in Health
SHI: social health inequalities
T2D: type 2 diabetes

## References

[1] Centers for Disease Control and Prevention (2016). Diabetes at a glance 2016.

[2] Nathan DM (2015). Diabetes: advances in diagnosis and treatment. JAMA.

[3] American Diabetes Association (2017). Standards of medical care in diabetes—2017: summary of revisions. Diabetes Care.

[4] Ali MK, Bullard KM, Gregg EW (2013). Achievement of goals in US diabetes care, 1999-2010. N Engl J Med.

[5] Public Law 111-5: Health Information Technology for Economic and Clinical Health (HITECH) of 2009.

[6] Public Law 111-148: The Patient Protection and Affordable Care Act of 2010.

[7] Office of the National Coordinator for Health Information Technology 2016 Report to Congress on health IT progress.

[8] Saparova D (2012). Motivating, influencing, and persuading patients through personal health records: a scoping review. Perspect Health Inf Manag.

[9] Martinez-Millana A, Fico G, Fernández-Llatas C, Traver V (2015). Performance assessment of a closed-loop system for diabetes management. Med Biol Eng Comput.

[10] Gutierrez M (2017). State telehealth laws and reimbursement policies.

[11] Latulippe K, Hamel C, Giroux D (2017). Social health inequalities and eHealth: a literature review with qualitative synthesis of theoretical and empirical studies. J Med Internet Res.

[12] Kaelber DC, Waheed R, Einstadter D, Love TE, Cebul RD (2013). Use and perceived value of health information exchange: one public healthcare system's experience. Am J Manag Care.

[13] Kaelber D (2015). MetroHealth core case study for Davies Award: return on investment financial value: historical ambulatory EHR ROI.

[14] Neuner J, Fedders M, Caravella M, Bradford L, Schapira M (2015). Meaningful use and the patient portal: patient enrollment, use, and satisfaction with patient portals at a later-adopting center. Am J Med Qual.

[15] Smith SG, O'Conor R, Aitken W, Curtis LM, Wolf MS, Goel MS (2015). Disparities in registration and use of an online patient portal among older adults: findings from the LitCog cohort. J Am Med Inform Assoc.

[16] Yamin C, Emani S, Williams D, Lipsitz S, Karson A, Wald J, Bates D (2011). The digital divide in adoption and use of a personal health record. Arch Intern Med.

[17] Perzynski A, Roach M, Shick S, Callahan B, Gunzler D, Cebul R, Kaelber D, Huml A, Thornton J, Einstadter D (2017). Patient portals and broadband Internet inequality. J Am Med Inform Assoc.

[18] Irizarry T, DeVito DA, Curran CR (2015). Patient portals and patient engagement: a state of the science review. J Med Internet Res.

[19] (2017). Digital inclusion outcomes-based evaluation.

[20] Hart JT (1971). The inverse care law. Lancet.

[21] Amante D, Hogan T, Pagoto S, English T (2014). A systematic review of electronic portal usage among patients with diabetes. Diabetes Technol Ther.

[22] Ancker JS, Barrón Y, Rockoff ML, Hauser D, Pichardo M, Szerencsy A, Calman N (2011). Use of an electronic patient portal among disadvantaged populations. J Gen Intern Med.

[23] Kruse RL, Koopman RJ, Wakefield BJ, Wakefield DS, Keplinger LE, Canfield SM, Mehr DR (2012). Internet use by primary care patients: where is the digital divide?. Fam Med.

[24] Ralston JD, Rutter CM, Carrell D, Hecht J, Rubanowice D, Simon GE (2009). Patient use of secure electronic messaging within a shared medical record: a cross-sectional study. J Gen Intern Med.

[25] US Bureau of the Census (2016). Types of Internet subscriptions by selected characteristics—American Community Survey 1-year estimates.

[26] Lewis J (2017). Handheld device ownership: reducing the digital divide?.

[27] Ancker J, Nosal S, Hauser D, Way C, Calman N (2017). Access policy and the digital divide in patient access to medical records. Health Policy and Technology.

[28] Smith A (2015). The smartphone difference.

[29] (2017). Bridging the gap: what affordable, uncapped Internet means for digital inclusion.

[30] Anderson M, Horrigan J Smartphones may not bridge digital divide for all.

[31] Gonzales A, Ems L, Suri V (2014). New Media Soc.

[32] Or CK, Tao D (2014). Does the use of consumer health information technology improve outcomes in the patient self-management of diabetes? A meta-analysis and narrative review of randomized controlled trials. Int J Med Inform.

[33] Steventon A, Bardsley M, Doll H, Tuckey E, Newman S (2014). Effect of telehealth on glycaemic control: analysis of patients with type 2 diabetes in the Whole Systems Demonstrator cluster randomised trial. BMC Health Serv Res.

[34] Hamine S, Gerth-Guyette E, Faulx D, Green B, Ginsburg A (2015). Impact of mHealth chronic disease management on treatment adherence and patient outcomes: a systematic review. J Med Internet Res.

[35] Krebs P, Duncan D (2015). Health app use among US mobile phone owners: a national survey. JMIR Mhealth Uhealth.

[36] Carroll J, Moorhead A, Bond R, LeBlanc W, Petrella R, Fiscella K (2017). Who uses mobile phone health apps and does use matter? A secondary data analytics approach. J Med Internet Res.

[37] Noblin A, Wan TT, Fottler M (2012). The impact of health literacy on a patient's decision to adopt a personal health record. Perspect Health Inf Manag.

[38] Sarkar U, Karter AJ, Liu JY, Adler NE, Nguyen R, Lopez A, Schillinger D (2010). The literacy divide: health literacy and the use of an Internet-based patient portal in an integrated health system-results from the diabetes study of northern California (DISTANCE). J Health Commun.

[39] Norman CD, Skinner HA (2006). eHealth literacy: essential skills for consumer health in a networked world. J Med Internet Res.

[40] Visser M Digital literacy definition.

[41] Kim H, Xie B (2017). Health literacy in the eHealth era: a systematic review of the literature. Patient Educ Couns.

[42] Tieu L, Schillinger D, Sarkar U, Hoskote M, Hahn KJ, Ratanawongsa N, Ralston JD, Lyles CR (2016). Online patient websites for electronic health record access among vulnerable populations: portals to nowhere?. J Am Med Inform Assoc.

[43] Tieu L, Sarkar U, Schillinger D, Ralston JD, Ratanawongsa N, Pasick R, Lyles CR (2015). Barriers and facilitators to online portal use among patients and caregivers in a safety net health care system: a qualitative study. J Med Internet Res.

[44] Lyles CR, Allen JY, Poole D, Tieu L, Kanter MH, Garrido T (2016). “I want to keep the personal relationship with my doctor”: understanding barriers to portal use among African Americans and Latinos. J Med Internet Res.

[45] Ackerman S, Sarkar U, Tieu L, Handley M, Schillinger D, Hahn K, Hoskote M, Gourley G, Lyles C (2017). Meaningful use in the safety net: a rapid ethnography of patient portal implementation at five community health centers in California. J Am Med Inform Assoc.

[46] Zhou YY, Garrido T, Chin HL, Wiesenthal AM, Liang LL (2007). Patient access to an electronic health record with secure messaging: impact on primary care utilization. Am J Manag Care.

[47] Kruse C, Argueta D, Lopez L, Nair A (2015). Patient and provider attitudes toward the use of patient portals for the management of chronic disease: a systematic review. J Med Internet Res.

[48] McAlearney A, Sieck C, Hefner J, Aldrich A, Walker D, Rizer M, Moffatt-Bruce S, Huerta T (2016). High touch and high tech (HT2) proposal: transforming patient engagement throughout the continuum of care by engaging patients with portal technology at the bedside. JMIR Res Protoc.

[49] Sarkar U, Karter AJ, Liu JY, Adler NE, Nguyen R, López A, Schillinger D (2011). Social disparities in internet patient portal use in diabetes: evidence that the digital divide extends beyond access. J Am Med Inform Assoc.

[50] Peacock S, Reddy A, Leveille SG, Walker J, Payne TH, Oster NV, Elmore JG (2017). Patient portals and personal health information online: perception, access, and use by US adults. J Am Med Inform Assoc.

[51] Reece J (2015). History matters: understanding the role of policy, race and real estate in today's geography of health equity and opportunity in Cuyahoga County.

[52] Callahan B AT&T's digital redlining of Cleveland.

[53] Strain G, Moore E, Gambhir S (2017). AT&Ts digital divide in California.

[54] Cebul R (2017). Celebrating 10 years of collaboration.

[55] Jones J, Weiner J, Shah N, Stewart W (2015). The wired patient: patterns of electronic patient portal use among patients with cardiac disease or diabetes. J Med Internet Res.

[56] Gibbons M, Casale CR (2010). Reducing disparities in health care quality: the role of health IT in underresourced settings. Med Care Res Rev.

[57] Aguilera A, Lyles C (2017). The case for jointly targeting diabetes and depression among vulnerable patients using digital technology. JMIR Diabetes.

[58] Gibbons M, Lowry S, Patterson E (2014). Applying human factors principles to mitigate usability issues related to embedded assumptions in health information technology design. JMIR Hum Factors.

[59] Fontil V, McDermott K, Tieu L, Rios C, Gibson E, Sweet C, Payne M, Lyles C (2016). Adaptation and feasibility study of a digital health program to prevent diabetes among low-income patients: results from a partnership between a digital health company and an academic research team. J Diabetes Res.

[60] Broderick A, Gillette D, Steinmetz V (2016). Digital health solutions to advance health and well-being for vulnerable populations.

[61] de Jong CC, Ros W, Schrijvers G (2014). The effects on health behavior and health outcomes of Internet-based asynchronous communication between health providers and patients with a chronic condition: a systematic review. J Med Internet Res.

[62] Jakicic J, Davis K, Rogers R, King W, Marcus M, Helsel D, Rickman A, Wahed A, Belle S (2016). Effect of wearable technology combined with a lifestyle intervention on long-term weight loss: the IDEA randomized clinical trial. JAMA.

[63] Lyles C, Sarkar U, Schillinger D, Ralston J, Allen J, Nguyen R, Karter A (2016). Refilling medications through an online patient portal: consistent improvements in adherence across racial/ethnic groups. J Am Med Inform Assoc.

[64] Shimada SL, Allison JJ, Rosen AK, Feng H, Houston TK (2016). Sustained use of patient portal features and improvements in diabetes physiological measures. J Med Internet Res.

[65] (2017). Everyone On.

[66] Ancker J (2017). Effective design and use of patient portals and their impact on patient-centered care.

[67] Bradlow Et, Hoch Sj, Wesley Hutchinson J (2002). An Assessment of Basic Computer Proficiency Among Active Internet Users: Test Construction, Calibration, Antecedents and Consequences. Journal of Educational and Behavioral Statistics.

[68] Northstar Basic Computer Skills Certificate.

[69] Chew LD, Bradley KA, Boyko EJ (2004). Brief questions to identify patients with inadequate health literacy. Fam Med.

[70] Sarkar U, Schillinger D, López A, Sudore R (2011). Validation of self-reported health literacy questions among diverse English and Spanish-speaking populations. J Gen Intern Med.

[71] US Bureau of the Census (2016). Types of computers and Internet subscriptions, 2015: American Community Survey 1-year estimates.

[72] Horrigan J, Duggan M (2015). Home broadband 2015.

[73] Bradlow Et, Hoch Sj, Wesley Hutchinson J (2002). An Assessment of Basic Computer Proficiency Among Active Internet Users: Test Construction, Calibration, Antecedents and Consequences. Journal of Educational and Behavioral Statistics.

[74] Center for Medicare & Medicaid Services Accountable Health Communities model.

[75] (2016). Using partnerships to power a smart city: a toolkit for local communities.

[76] National Telecommunications & Information Administration.

[77] Schartman S (2012). Connect Your Community participant survey results.

[78] Schartman-Cycyk S (2017). The digital divide: literacy, access and equity. Cross-Sector Panel: Health Digital Divide.

[79] Institute for Medicaid Innovation (2017). Medicaid managed care best practices compendium 2016-2017.

[80] (2016). FCC modernizes lifeline program for the digital age.

[81] Davis W (2016). National Digital Inclusion Alliance.

[82] Harris K (2016). National Digital Inclusion Alliance.

[83] Lyles C, Tieu L, Kiyoi S (2016). National Digital Inclusion Alliance.

[84] Sheon A (2016). Community digital inclusion partnerships with healthcare providers: panel discussion.

[85] Sheon A (2017). The ROI of digital inclusion for community health and health care.

[86] Dietz W, Belay B, Bradley D, Kahan S, Murth N, Sanchez E, Solomon L (2017). A model framework that integrates community and clinical systems for the prevention and management of obesity and other chronic diseases.

[87] Barton J (2016). Closing the digital divide: a framework for meeting CRA obligations.

[88] Greysen S, Magan MY, Rosenthal J, Jacolbia R, Rajkomar A, Lee H, Auerbach A (2016). Using tablet computers to increase patient engagement with electronic personal health records: protocol for a prospective, randomized interventional study. JMIR Res Protoc.

[89] Sadasivaiah S, Kiyoi S, Ratanawongsa N, Lyles C (2016; 11 (suppl 1).). Finding meaning in meaningful use: implementing a patient portal in an urban safety net academic hospital. J Hosp Med.

[90] Druss B, Ji X, Glick G, von Esenwein SA (2014). Randomized trial of an electronic personal health record for patients with serious mental illnesses. Am J Psychiatry.

[91] Jacobs G, Castek J, Pizzolato D, Pendell K, Withers E, Reder S (2015). Executive summary: tutor-facilitated digital literacy acquisition.

[92] Zikmund-Fisher B, Exe N, Witteman H (2014). Numeracy and literacy independently predict patients' ability to identify out-of-range test results. J Med Internet Res.

[93] Sarkar U, Bates D (2014). Care partners and online patient portals. JAMA.

[94] Celi L, Davidzon G, Johnson A, Komorowski M, Marshall D, Nair S, Phillips C, Pollard T, Raffa J, Salciccioli J, Salgueiro F, Stone D (2016). Bridging the health data divide. J Med Internet Res.

[95] Horowitz C, Shameer K, Gabrilove J, Atreja A, Shepard P, Goytia C, Smith G, Dudley J, Manning R, Bickell N, Galvez M (2017). Accelerators: sparking innovation and transdisciplinary team science in disparities research. Int J Environ Res Public Health.

[96] Sheon A (2016). Building digital evidence together to fast track technologies that improve patient outcomes. http://exhibitionfloor.himss.org/chc2016/Public/SessionDetails.aspx?FromPage=Sessions.aspx&SessionID=1260&SessionDateID=19.

[97] Sarkar U, Gourley GI, Lyles CR, Tieu L, Clarity C, Newmark L, Singh K, Bates DW (2016). Usability of commercially available mobile applications for diverse patients. J Gen Intern Med.

[98] Makhni S, Atreja A, Sheon A, Van WB, Sharp J, Carpenter N (2017). The broken health information technology innovation pipeline: a perspective from NODE Health Consortium. Digital Biomarkers.

[99] Modave F, Bian J, Rosenberg E, Mendoza T, Liang Z, Bhosale R, Maeztu C, Rodriguez C, Cardel M (2016). DiaFit: The Development of a Smart App for Patients with Type 2 Diabetes and Obesity. JMIR Diabetes.

[100] Perzynski A The Patient-Centered Media Lab.

[101] Perzynski A, Johnson E, Schick S, Smith T (2016). HealthStead: home is where the health is.

[102] (2017). Mobile fact sheet.

[103] Levy N, Moynihan V, Nilo A, Singer K, Bernik L, Etiebet M, Fang Y, Cho J, Natarajan S (2015). The mobile insulin titration intervention (MITI) for insulin adjustment in an urban, low-income population: randomized controlled trial. J Med Internet Res.

[104] Thakkar J, Kurup R, Laba T, Santo K, Thiagalingam A, Rodgers A, Woodward M, Redfern J, Chow C (2016). Mobile telephone text messaging for medication adherence in chronic disease: a meta-analysis. JAMA Intern Med.

